# Positive and negative affect and oral health-related quality of life

**DOI:** 10.1186/1477-7525-4-83

**Published:** 2006-10-20

**Authors:** David S Brennan, Kiran A Singh, A John Spencer, Kaye F Roberts-Thomson

**Affiliations:** 1 Australian Research Centre for Population Oral Health, School of Dentistry, The University of Adelaide, South Australia 5005 Australia

## Abstract

**Background:**

The aims of the study were to assess the impact of both positive (PA) and negative affect (NA) on self-reported oral health-related quality of life and to determine the effect of including affectivity on the relationship between oral health-related quality of life and a set of explanatory variables consisting of oral health status, socio-economic status and dental visiting pattern.

**Methods:**

A random sample of 45–54 year-olds from metropolitan Adelaide, South Australia was surveyed by mailed self-complete questionnaire during 2004–05 with up to four follow-up mailings of the questionnaire to non-respondents (n = 986 responded, response rate = 44.4%). Oral health-related quality of life was measured using OHIP-14 and affectivity using the Bradburn scale. Using OHIP-14 and subscales as the dependent variables, regression models were constructed first using oral health status, socio-economic characteristics and dental visit pattern and then adding PA and NA as independent variables, with nested models tested for change in R-squared values.

**Results:**

PA and NA exhibited a negative correlation of -0.49 (P < 0.01). NA accounted for a larger percentage of variance in OHIP-14 scores (3.0% to 7.3%) than PA (1.4% to 4.6%). In models that included both PA and NA, PA accounted for 0.2% to 1.1% of variance in OHIP-14 scores compared to 1.8% to 3.9% for NA.

**Conclusion:**

PA and NA both accounted for additional variance in quality of life scores, but did not substantially diminish the effect of established explanatory variables such as oral health status, socio-economic status and dental visit patterns.

## Background

Health-related quality of life is a multidimensional concept that includes patient-driven measures such as perceptions and functional status [1]. Oral health-related quality of life measures emerged out of the development of socio-dental indicators to capture non-clinical aspects of oral health that broadened the focus of oral epidemiological research [2].

Self-reported health measures have been demonstrated to reflect a pervasive mood disposition of negative affectivity [3]. Negative affectivity (NA) has been described as a general disposition to experience negative mood states, and has been found to be consistently negatively related to health-related quality of life indicating that personality as well as underlying health can influence self-ratings of health-related quality of life [4]. NA has also been related to oral health-related quality of life, with higher negative affect consistently associated with worse scores on oral health-related quality of life measures [5].

While affectivity has typically been measured in terms of NA, it is also possible to measure positive affect (PA). The terms PA and NA are not considered as opposites, but as distinctive dimensions with high PA a state of high energy, full concentration and pleasurable engagement and low NA a state of calmness and serenity [6]. Given the distinct nature of PA and NA it is important to distinguish their respective potential influences on self-ratings of health-related quality of life. A range of other factors has also been related to oral health-related quality of life, including oral health status, dental visit pattern, socio-demographics and socio-economic status. Differences in prevalence of social impact have been reported for oral health factors such as between dentate and edentulous persons [7], and numbers of missing teeth [8]. Dental attendance has been associated with the perception of an enhanced quality of life [9], as well as other dental visit factors such as place of visit [10], and reason for visit [8,11]. Specific dental interventions that have been associated with improved oral health-related quality of life include implant-retained dental prostheses [12-15], conventional fixed prosthodontics [16,17], third molar removal [18], orthodontic treatment [19], orthognathic surgery [20-22], occlusal splints in therapy for temporomandibular disorder [23], and surgery for oral cancer [24]. Socio-demographic and socio-economic factors related to oral health-related quality of life include age and cultural background [25], as well as gradients in oral conditions in relation to social status [26]. The aims of the study were to assess the impact of both PA and NA on self-reported oral health-related quality of life and to determine the effect of including affectivity on the relationship between oral health-related quality of life and a set of explanatory variables consisting of oral health status, socio-economic status and dental visiting pattern so as to determine the independent effect of PA and NA when adjusting for a set of established explanatory variables.

## Methods

### Sampling and data collection

A total of 2469 persons aged 45–54 years were randomly sampled from metropolitan Adelaide, South Australia, using the Electoral Roll as a sampling frame. Sampled persons were surveyed by mailed self-complete questionnaire during 2004-05. A primary approach letter was initially mailed, followed a week later by the questionnaire, and then by a reminder card and up to four follow-up mailings of the questionnaire to non-respondents in order to achieve a higher response rate [27].

### Variables measured

A range of variables were collected spanning self-reported number of teeth, dental visiting, dental behaviours, socio-demographics, socio-economic status and psycho-social variables. Oral health-related quality of life was measured using the OHIP-14 instrument [10]. The Oral Health Impact Profile (OHIP) is a disease-specific measure of people's perceptions of the social impact of oral disorders on their well-being [28]. OHIP contains 49 questions that capture seven conceptually formulated dimensions based on Locker's theoretical model of oral health [29], and the OHIP-14 was developed as a shorter version of the OHIP for settings where the full battery is inappropriate [10]. Subjects were asked to rate their experience of dental problems in the last year using the OHIP-14. For each of the OHIP-14 questions subjects were asked how frequently they had experienced an impact in the preceding 12 months using a Likert-type response scale coded as 0 = never, 1 = hardly ever, 2 = occasionally, 3 = fairly often and 4 = very often. OHIP sub-scale scores were produced by applying the published item weights and then summing the responses to the 2 weighted items for each sub-scale [10]. Note that the item weights sum to 1.0 for each sub-scale, so that weighted scores will have a smaller range than if they were unweighted. The seven dimensions of OHIP are functional limitation, physical pain, psychological discomfort, physical disability, psychological disability, social disability and handicap. The total OHIP scale score was produced by summing the weighted sub-scale scores. Affectivity was measured using the Bradburn scale of positive and negative affect [30]. The 18 items consisted of 9 items measuring PA and 9 items measuring NA that asked how often each item occurred during the last year using a 5-point scale coded as 0 = never, 1 = hardly ever, 2 = occasionally, 3 = fairly often and 4 = very often. Scores for both PA and NA were produced by summing the responses to the 9 items for each scale.

### Analysis

Representativeness of the sample respondents was assessed by comparison to a range of oral health status, sociodemographic and dental visiting pattern variables from a population survey [31]. OHIP-14 scores formed the main dependent variable in regressions which were analysed by comparing the change in R-squared values between nested models [32], comprising model 1 (independent variables of oral health status, socio-economic status and dental visiting pattern), model 2 (independent variables of oral health status, socio-economic status and dental visit pattern plus PA), model 3 (independent variables of oral health status, socio-economic status and dental visiting pattern plus NA), and model 4 (independent variables of oral health status, socio-economic status and dental visiting pattern plus both PA and NA). All models included number of teeth as a continuous variable, card holder status as a socio-economic indicator variable coded as 1 for card holders (such as unemployed and aged pensioners) and 0 for no card, and reason for visit as an indicator variable coded as 1 if the last dental visit was for relief of pain and 0 if not for relief of pain. PA and NA scales were included as continuous variables.

The set of explanatory variables included in the models was determined on the basis of producing a parsimonious model that covered a range of subject domains (i.e., oral health, visit pattern, socio-economic status). In doing so variables were selected to represent each subject domain on the basis that they had dental relevance through their common inclusion in other studies, and consistent and strong associations with dental service provision, analytic applicability through minimal missing data and limited collinearity, and policy relevance to dental public health. Number of teeth was included as this variable has been associated with oral health related quality of life [8,10,33], and was considered more appropriate than denture wearing, which has a relatively low prevalence in this age group. While demographic variables are not included directly in the models, the study population is restricted in age to 45–54 year-olds and in geographic location to metropolitan Adelaide, South Australia. Card holder status was selected over income to represent socio-economic status on the basis of minimising missing data, as income may suffer non-response bias due to sensitivity in disclosure [34], and due to the policy relevance of card holder status. In Australia, health concession card holders represent a low-income group who may be eligible for public dental care. Dental visit pattern was represented by the variable of last dental visit being for relief of pain, as this variable has shown consistent and strong associations with provision of dental services [35-38].

## Results

### Response

A total 986 persons responded giving a response rate of 44.4% after adjusting for out of scope persons such as those who could not be contacted. The study participants generally showed a close approximation of the population profile (Table 1). Study participants had slightly lower numbers of teeth, but there was no difference in denture wearing. Study participants had a slightly lower percentage visiting in the last 12 months and slightly fewer numbers of visits in the last 12 months, as well as a lower percentage that visited privately at the last visit, but there was no difference in the percentage receiving check-ups at the last dental visit. There were no differences in the percentage of females, Australian-born, or Indigenous status, but study participants had a slightly higher percentage who spoke English as the main language at home as well as a slightly higher percentage who were concession card holders, but there was no difference in the percentage of persons from higher income households.

**Table 1:** Comparison of study participants with population profile

	*Population	**Study participants**	
**Oral health status**			(95% CI)
Number of teeth – mean	26.9	25.4	(25.0–25.8)
Denture (upper jaw) – %	13.7	13.5	(11.4–15.7)
Denture (lower jaw) – %	5.8	6.4	(4.7–7.9)
			
**Dental visit pattern**			
Last dental visit <12 months – %	65.4	61.5	(58.4–64.5)
Check-up at last dental visit – %	41.7	42.8	(39.7–45.9)
Number of dental visits in last 12 months – mean	1.8	1.5	(1.4–1.7)
Visited private at last dental visit – %	95.2	86.4	(84.3–88.6)
			
**Socio-demographics**			
Female sex – %	51.2	52.9	(49.8–56.0)
Australian born – %	70.8	71.3	(68.4–74.2)
Indigenous – %	1.3	0.4	(0–6.8)
English main language at home – %	91.9	95.4	(94.1–96.7)
			
**Socio-economic status**			
Concession card holder – %	15.4	18.0	(15.6–20.4)
Household income $80,000+ – %	24.5	23.9	(21.2–26.7)

*National Dental Telephone Interview Survey 2002: South Australia – Adelaide 45–54 year-olds

### Distribution of scale items

The distribution of responses to the OHIP scale items is presented in Table 2. The majority of responses to the items were in the category of 'never', with the exception of the two items from the physical pain sub-scale. The two items from the psychological discomfort sub-scale also had showed relatively high levels of problems compared to the remaining items. The distribution of responses to the affectivity scale items is presented in Table 3. Generally, there were a high percentage of responses in the 'occasionally' category for all items. The items comprising the positive affect scale also had a high percentage of responses in the 'fairly often' category. The items comprising the negative affect scale tended to have a high percentage of responses in the 'hardly ever' category, with the exception of the items 'annoyed with someone' and 'really tired', which had more responses in the 'fairly often' than the 'hardly ever' categories.

**Table 2:** Distributions of responses to OHIP scale items (%)

How often in the past year have you had the following problems?	**Never**	**Hardly ever**	**Occasionally**	**Fairly often**	**Very often**
**Functional limitation**					
trouble pronouncing any words	83.8	8.4	5.7	1.4	0.7
sense of taste has worsened	81.6	8.6	6.9	1.7	1.1
**Physical pain**					
painful aching in your mouth	49.3	25.4	21.2	2.6	1.4
uncomfortable to eat any foods	43.5	23.9	24.9	3.6	4.1
**Psychological discomfort**					
felt self conscious	51.4	16.6	17.1	7.7	7.1
felt tense	61.9	16.6	13.8	4.6	3.1
**Physical disability**					
diet been unsatisfactory	81.3	11.7	4.3	1.9	0.8
had to interrupt meals	74.6	15.3	7.7	1.7	0.7
**Psychological disability**					
found it difficult to relax	71.9	16.2	8.5	2.1	1.2
been a bit embarrassed	64.1	13.2	15.3	3.4	4.0
**Social disability**					
been a bit irritable with other people	78.5	11.7	7.6	1.1	1.1
had difficulty doing your usual jobs	86.5	9.2	3.6	0.5	0.2
**Handicap**					
life in general was less satisfying	75.6	11.5	9.2	1.8	2.0
been totally unable to function	90.5	6.7	2.2	0.4	0.3

**Table 3:** Distributions of responses to affectivity scale items (%)

During the last year how often have you felt...	**Never**	**Hardly ever**	**Occasionally**	**Fairly often**	**Very often**
annoyed with someone?*	0.9	10.5	57.9	22.7	8.0
very lonely or remote from other people?*	12.5	38.1	34.3	9.4	5.6
that things were going your way?	1.4	9.1	35.9	46.7	6.9
very worried?*	1.9	21.4	46.7	20.6	9.4
pleased because you've got good friends?	1.4	8.6	28.2	39.4	22.5
afraid of what might happen?*	4.8	30.8	41.0	15.3	8.2
particularly excited or interested in something?	0.6	5.8	39.6	42.7	11.3
depressed or very unhappy?*	9.5	36.1	36.8	10.5	7.1
full of energy?	0.8	11.4	37.3	42.7	7.9
really tired?*	0.8	10.8	47.4	29.9	11.1
so restless that you couldn't sit long in a chair?*	10.0	38.0	33.9	13.0	5.1
that you were really enjoying yourself?	0.6	6.8	38.1	45.7	8.7
really cheerful?	0.6	5.3	34.7	49.7	9.7
like crying?*	7.8	43.0	37.4	8.1	3.8
on top of the world?	4.5	19.6	46.7	25.9	3.4
confident about the future?	2.8	12.3	35.0	42.7	7.2
bored?*	13.1	34.8	37.0	12.2	2.9
pleased about having accomplished something?	0.7	3.9	37.8	48.7	8.9

*items 1,2,4,6,8,10,11,14,17 measure negative affect, the remaining items measure positive affect

### Distribution of key continuous variables

Table 4 shows the overall OHIP-14 scale had a mean ± SD of 3.55 ± 4.50, with subscales ranging from 0.29 ± 0.64 for functional limitation to 0.94 ± 0.93 for pain/discomfort, with higher scores indicating a greater number of impacts on quality of life. PA had a higher value (22.41 ± 5.51) compared to NA (16.73 ± 5.75), indicating a perception of more frequent positive than negative feelings. Note that PA and NA exhibited a negative correlation of -0.49 (P < 0.01).

**Table 4:** Distribution of OHIP and affectivity variables

	**Mean**	**S.D.**	**Min.**	**Max.**
**OHIP subscale**				
Functional limitation	0.29	0.64	0.0	4.0
Physical pain	0.94	0.93	0.0	4.0
Psychological discomfort	0.85	1.07	0.0	4.0
Physical disability	0.34	0.67	0.0	4.0
Psychological disability	0.55	0.84	0.0	4.0
Social disability	0.29	0.62	0.0	4.0
Handicap	0.31	0.65	0.0	4.0
**OHIP scale**				
OHIP score	3.55	4.50	0.0	25.7
**Affectivity variables**				
Positive affect	22.41	5.51	0.0	36.0
Negative affect	16.73	5.75	2.0	36.0

### Bivariate associations

Table 5 shows bivariate associations between OHIP scale and subscale scores and explanatory variables covering oral health status, socio-economic status, dental visiting pattern, and affectivity. Numbers of teeth and PA both showed consistent negative correlations with OHIP scale and subscale scores, while NA had consistent positive correlations with OHIP scale and subscale scores. Card holders had higher scores for all subscales and the overall OHIP scale compared to those did not have a concession card. If the last dental visit was made for relief of pain, scores on all subscales and the overall OHIP scale were higher compared to those who had made their last visit for reasons other than relief of pain.

**Table 5:** Bivariate relationships between OHIP and explanatory variables

	***Functional limitation***	***Physical pain***	***Psychol. discomfort***	***Physical disability***	***Psychol. disability***	***Social disability***	***Handicap***	***OHIP scale***
	***rho***	***rho***	***rho***	***rho***	***rho***	***rho***	***rho***	***rho***

Number of teeth	**-0.33	**-0.25	**-0.29	**-0.30	**-0.29	**-0.17	**-0.24	**-0.32
Positive affect	**-0.22	**-0.27	**-0.28	**-0.27	**-0.30	**-0.26	**-0.29	**-0.33
Negative affect	**0.27	**0.28	**0.31	**0.30	**0.35	**0.28	**0.30	**0.36

	***Mean (SE)***	***Mean (SE)***	***Mean (SE)***	***Mean (SE)***	***Mean (SE)***	***Mean (SE)***	***Mean (SE)***	***Mean (SE)***

Card status	**	**	**	**	**	**	**	**
Card holder	0.56 (.07)	1.35 (.08)	1.40 (.01)	0.72 (.07)	1.03 (.09)	0.53 (.06)	0.67 (.07)	6.23 (.46)
Not card holder	0.23 (.02)	0.85 (.03)	0.72 (.03)	0.25 (.02)	0.44 (.03)	0.23 (.02)	0.22 (.02)	2.92 (.13)
Reason for visit	**	**	**	**	**	**	**	**
Relief of pain	0.47 (.07)	1.42 (.09)	1.27 (.10)	0.63 (.07)	0.92 (.08)	0.56 (.07)	0.54 (.07)	5.78 (.46)
Not relief of pain	0.25 (.02)	0.85 (.03)	0.76 (.04)	0.28 (.02)	0.47 (.03)	0.23 (.02)	0.26 (.02)	3.10 (.14)

**(P < 0.01)

### OHIP scale models

Table 6 presents models for OHIP scale scores by oral health status, socio-economic status, dental visiting pattern and affectivity. In each model number of teeth had negative coefficients indicating more teeth were associated with lower numbers of oral health impacts, and holding a health card and visiting for relief of pain both had positive coefficients indicating they were associated with higher numbers of oral health impacts. PA had negative coefficients indicating an association with lower numbers of oral health impacts, while NA had positive coefficients indicating an association with higher numbers of oral health impacts. Comparison of regression coefficients across models indicated that the addition of affectivity tended to attenuate the effects of oral health status, socio-economic status, and dental visiting pattern. This attenuation was only slight in the case of number of teeth, but was more pronounced for card holder status and a dental visit pattern of relief of pain visits. PA and NA also tended to be attenuated in the presence of each other, but this attenuation was more pronounced for PA when NA was included.

**Table 6:** Regression coefficients from models of OHIP scale scores

	**Model 1**	**Model 2**	**Model 3**	**Model 4**
**Variables**				
Number of teeth	**-0.19	**-0.19	**-0.18	**-0.18
Card holder (ref. No card)	**2.58	**1.99	**1.81	**1.64
Relief of pain visit (ref. not relief of pain)	**2.05	**1.74	**1.81	**1.69
Positive affect	-	**-0.17	-	**-0.09
Negative affect	-	-	**0.21	**0.17
**Model statistics**				
P value	<0.0001	<0.0001	<0.0001	<0.0001
R-sq	0.2022	0.2489	0.2757	0.2862
**R-sq change**				
(model 2 vs model 1)	-	**0.0467	-	-
(model 3 vs model 1)	-	-	**0.0735	-
(model 4 vs model 2)	-	-	-	**0.0373
(model 4 vs model 3)	-	-	-	**0.0105

**(P < 0.01)

In model 1, which included oral health status, socio-economic status, and dental visiting pattern the overall R-squared value was 20.2%. The addition of PA (model 2) contributed an additional 4.7% increase in R-squared over model 1, while the addition of NA (model 3) contributed an additional 7.4% increase in R-squared over model 1. The addition of PA (model 4) to the model already containing NA (model 3) contributed to a 1.1% increase in R-squared while addition of NA (model 4) to the model already containing PA (model 2) contributed to a 3.7% increase in R-squared value.

When expressed as a relative increase, the additional R-squared of 0.0467 obtained from adding PA (model 2) to the model containing oral health status, socio-economic status, and dental visiting pattern (model 1) which had an R-squared of 0.2022 provided a 23.1% increase in R-squared, while the additional R-squared of 0.0735 obtained from adding NA (model 3) to the R-squared of 0.2022 observed for model 1 provided a 36.4% increase in the R-squared value.

### OHIP subscale models

Table 7 presents R-squared values from models for OHIP subscale scores by oral health status, socio-economic status, dental visit pattern and affectivity. Note that across all 7 subscales and all 4 models of each subscale there were consistent negative coefficients for number of teeth, and positive coefficients for card holder status and reason for visit. For each subscale there was some attenuation of effect in strength of coefficients for card holder status and reason for visit when comparing simpler models with more complex models (i.e., moving from model 1 through to model 4), but each effect remained statistically significant. The addition of PA (model 2 versus model 1) contributed to a significant increase of between 1.4% and 3.9% in amount of variance explained for all subscales, as did the addition of NA (model 3 versus model 1), which added between 3.0% and 6.9%. In models that included both PA and NA, the addition of PA (model 4 versus model 3) contributed to a significant increase in 6 of the 7 subscales of between 0.5% and 1.1%, while the addition of NA (model 4 versus model 2) contributed to a significant increase in variance explained in all 7 subscales of between 1.8% and 3.9%.

**Table 7:** Summary of R-squared values from regression models of OHIP subscale scores

	**Model 1**	**Model 2**	**Model 3**	**Model 4**
**OHIP subscale used as dependent variable**				
Functional limitation	0.1494	^(a)^0.1634	^(b)^0.1793	^(c)^0.1815
Physical pain	0.1366	^(a)^0.1690	^(b)^0.1778	^(c, d)^0.1879
Psychological discomfort	0.1539	^(a)^0.1885	^(b)^0.2080	^(c, d)^0.2166
Physical disability	0.1747	^(a)^0.1977	^(b)^0.2166	^(c, d)^0.2212
Psychological disability	0.1704	^(a)^0.2082	^(b)^0.2396	^(c, d)^0.2472
Social disability	0.0856	^(a)^0.1139	^(b)^0.1359	^(c, d)^0.1419
Handicap	0.1264	^(a)^0.1637	^(b)^0.1756	^(c, d)^0.1868

Note: model 1 independent variables include number of teeth, card holder status, relief of pain visit, model 2 includes number of teeth, card holder status, relief of pain visit plus positive affect, model 3 includes number of teeth, card holder status, relief of pain visit plus negative affect, and model 4 includes number of teeth, card holder status, relief of pain visit plus both positive affect and negative affect.

(a): model 2 vs model 1 (P < 0.05)

(b): model 3 vs model 1 (P < 0.05)

(c): model 4 vs model 2 (P < 0.05)

(d): model 4 vs model 3 (P < 0.05)

## Discussion

### Representativeness

While the overall response yield of n = 986 provided sufficient numbers for analysis, the response rate was lower than anticipated. This was particularly so, since multiple follow-ups were employed to increase the response rate as per the Total Design Method [27]. The use of the Electoral Roll should provide an adequate sampling frame for a population survey of 45–54 year-olds. Bias can distort the design, execution, analysis and interpretation of research [39]. Sampling bias is unlikely, since voting is compulsory for adults in Australia and the sample was drawn at random, the Electoral Roll this should provide a representative sampling frame. Although it is possible that some sub-groups of the population (eg. the homeless) would be under-enumerated, such sub-groups are not the focus of the study. Also the use of restriction can limit selection bias in the design of a study [40], and a restricted age range was adopted in this study. Generally, a response rate of 60% is considered adequate [41], with lower response rates requiring evidence to determine whether bias has been introduced. The issue is whether a lower response rate involves differential response among population sub-groups that could produce bias.

While direct comparison of respondents and non-respondents would be desirable to assess response bias we were only able to compare the profile of respondents with population data. This comparison showed generally small differences between the population data and the study participants based on the range of variables that were compared, the main difference observed being the lower percentage of survey respondents that visited privately at the last dental visit compared to the population consistent with the slightly higher percentage who were concession card holders. Since card holders are eligible for public dental care this is also consistent with observed slightly lower numbers of teeth, lower percentage visiting in the last 12 months, and fewer numbers of visits in the last 12 months. Selection bias can be controlled in the analysis of a study through adjustments such as stratification or multivariate analysis [40]. Hence, card holder status was included in the statistical models along with two key oral health and visit variables, number of teeth and reason for visit.

While the relatively low response rate did not appear to produce bias in the respondents compared with the population, it raises the issue of the difficulty in achieving high response rates in population studies. Data from the USA has shown a trend toward substantial increases in total non-response, primarily due to increased percentages of respondents who refused to be interviewed that was related to the level of urbanisation [42]. However, response rate is considered only an indirect indication of the extent of non-response bias, and more attention is required to assessments of bias rather than to specific response rate thresholds [43]. It should be noted that although the response was relatively low in this study, it was obtained using the Total Design Method [27], incorporating aspects such as repeated contacts and return postage that have been shown to increase response behaviour [44].

### Oral health, visit pattern and socio-economic status

While PA and NA were the focus of the study, the set of additional explanatory variables included in the models (i.e., oral health, visit pattern, socio-economic status) warrant some discussion. Regardless of the inclusion of PA or NA, significant effects were observed between oral health-related quality of life and number of teeth, card holder status and dental visit pattern. The negative relationship between number of teeth and worsening oral health-related quality of life has been observed previously [8,10], and underscores the importance of tooth retention. The positive association between card holder status and poorer oral health-related quality of life is consistent with previous observations that card holders in Australia suffer from problems with access to dental care, and these access problems have been associated with high levels of emergency visits, and high rates of extraction [45]. Problem-oriented dental visit patterns have been associated with less favourable patterns of dental service provision in the private [38] and public sector [46], poorer oral health status [47], and worse quality of life [33]. Similar observations have been made in other countries, such as higher recovery from quality of life decrements among regular dental attenders and those making check-up visits in the USA [48].

### Positive and negative affect

PA and NA have been found to relate to different classes of variables, with NA (but not PA) related to stress and coping, health complaints and frequency of unpleasant events, and PA (but not NA) related to social activity and satisfaction, and frequency of pleasant events [6]. While PA and NA are considered separate dimensions, and both are associated with quality of life in the expected directions and in the presence of each other – the influence of PA was attenuated in the presence of NA, indicating the more dominant role for NA in relation to quality of life. The attenuation may be expected given the observed correlation between PA and NA. While the observed correlation was in the range of low to moderate [49], this size of effect is unlikely to result in collinearity problems. Collinearity refers to relationships among the independent variables and is used to indicate that one predictor is an exact linear combination of the others. Near collinearity arises when there is a high degree of association between independent variables and may result in inaccurate estimates of regression coefficients, standard errors and hypothesis test statistics [32].

Not all studies find such correlations between PA and NA and this most likely reflects the adoption of a longer time frame of "last year" with the Bradburn scale in this study, as affectivity scales have been reported to be more reliable over longer time frames [6], such that the scales should represent relatively fixed personality dispositions rather than recent life events [30]. The relationship of personality, particularly negative affect, and well-being has been explained through a dynamic equilibrium model involving both personality and life events [50], whereby life events may alter well-being temporarily before personality traits draw people back to their usual level of life events and well-being. Other studies looking at cognitive functioning have found NA to be more strongly related to self-appraisal of cognitive functioning than PA [51]. Affectivity in terms of PA and NA has been suggested as a mediator of the link between outcome expectancies related to optimism and pessimism and psychological adjustment in terms of depressive symptoms and life satisfaction [52]. Self-esteem has also been shown to be strongly negatively correlated with neuroticism/NA and moderately to strongly related to extraversion/PA [53].

### Subjectivity of quality of life measures and affectivity

It may be expected that more subjective indices such as the OHIP subscales psychological discomfort and psychological disability would have stronger associations with NA than less subjective indices such as physical disability and functional limitations [5]. In this study NA either alone (model 3) or in the presence of PA (model 4) contributed the least amount of additional variance in OHIP-14 scores for the less subjective functional limitation subscale and the most for the more subjective psychological disability subscale. In the absence of NA (model 2) PA also contributed the least amount of additional variance for the functional limitation subscale and the most for the psychological disability subscale. In the presence of NA (model 4) PA showed little change in additional variance between subscales, but did not contribute any significant additional variance to the functional limitation subscale. As previously reported this provides some support for the hypothesis that more subjective indices are more strongly influenced by affectivity [5], including PA, but that the influence of PA on quality of life measures is attenuated when controlling for NA.

### Symptom perception

One main path of symptom perception of common physical symptoms has been identified as involving more NA via a stronger tendency to selective attention [54]. A study of chronically ill patients found that optimism did not tend to bias their perceptions of their health status but that positive efficacy expectancies encouraged self-care behaviour [55]. Another study of self-reported symptoms among asthmatics found that patients with high NA were more influenced by suggestion than patients with low NA [56]. However, the tendency to overestimate the likelihood of ambiguous symptoms as being indicative of serious illness appears to be unique to hypochondriasis and not attributable to high NA [57].

While experience of oral symptoms may vary between individuals, some specific oral symptoms such as toothache are considered more likely to influence quality of life and alter behaviour through the seeking of professional care than other symptoms such as sore gums or sensitive teeth [58]. There is also evidence of specific relationships between oral health status and reported impact on quality of life, such as number of teeth and chewing ability, missing front teeth with self-esteem and going out, as well as fewer functioning teeth and more decayed teeth with aesthetic dissatisfaction, altered eating, diminished communication and pain [58].

Personality, through affectivity, may influence quality of life measures through symptom perception, whereby high NA individuals are more sensitive to health conditions and therefore more likely to perceive and/or complain about health concerns resulting in worse quality of life scores and possibly inflated health-related complaints [5]. More subjective indices of quality of life may therefore be more prone to being influenced by personality variables such as affectivity. This may warrant the need to control for personality factors, but also provides additional insight into the understanding how health conditions influence functioning and well-being [5]. There is also evidence that positive mental states are more than the absence of symptoms, and may play an independent role in health outcomes [59].

## Conclusion

PA and NA both accounted for additional variance in quality of life scores indicating that personality factors have independent effects on self-ratings of health-related quality of life. Futhermore, the addition of both PA and NA revealed some attenuation of the effects of oral health status, socio-economic status, and dental visiting pattern indicating that some of the association of these variables with health-related quality of life reflects underlying mood states. However, PA and NA did not substantially diminish the effect of established explanatory variables such as oral health status, socio-economic status and dental visiting pattern confirming their independent effects on oral health-related quality of life.

## Authors' contributions

DSB, AJS and KFRT were chief investigators on the grant obtained to fund the study, and participated in the design and coordination of the study. DSB and KAS performed the analysis and drafting of the manuscript. AJS and KFRT assisted with the completion of the manuscript. All authors read and approved the manuscript.
